# A nanometric Rh overlayer on a metal foil surface as a highly efficient three-way catalyst

**DOI:** 10.1038/srep29737

**Published:** 2016-07-08

**Authors:** Satoshi Misumi, Hiroshi Yoshida, Satoshi Hinokuma, Tetsuya Sato, Masato Machida

**Affiliations:** 1 Department of Applied Chemistry and Biochemistry, Graduate School of Science and Technology, Kumamoto University, Kumamoto, 860-8555, Japan; 2 Unit of Elements Strategy Initiative for Catalysts & Batteries, Kyoto University, Kyoto, 615-8245, Japan; 3 Precursory Research for Embryonic Science and Technology, Japan Science and Technology Agency, Saitama, 332-0012, Japan; 4 Technical Division, Faculty of Engineering, Kumamoto University, Kumamoto, 860-8555, Japan

## Abstract

Pulsed arc-plasma (AP) deposition of an Rh overlayer on an Fe–Cr–Al stainless steel foil produced a composite material that exhibited high activity for automotive three-way catalysis (TWC). The AP pulses deposited metallic Rh nanoparticles 1–3 nm in size, whose density on the surface increased with the number of pulses. This led to coalescence and grain growth on the foil surface and the eventual formation of a uniform two-dimensional Rh overlayer. Full coverage of the 51 μm-thick flat foil by a 3.2 nm-thick Rh overlayer was achieved after 1,000 pulses. A simulated TWC reaction using a miniature honeycomb fabricated using flat and corrugated foils with the Rh overlayers exhibited successful light-off at a practical gaseous hourly space velocity of 1.2 × 10^5^ h^−1^. The turnover frequency for the NO–CO reaction over the metallic honeycomb catalyst was ca. 80-fold greater than that achieved with a reference Rh/ZrO_2_-coated cordierite honeycomb prepared using a conventional wet impregnation and slurry coating procedure. Despite the nonporosity and low surface area of the foil-supported Rh overlayer compared with conventional powder catalysts (Rh/ZrO_2_), it is a promising alternative design for more efficient automotive catalysts that use less Rh loading.

The exhaust gases from gasoline-fuelled automobiles are typically purified using a three-way catalyst (TWC) comprising nanoparticles of a platinum-group metal (PGM), a metal oxide support (or ‘washcoat’) containing an oxygen storage component and a monolithic honeycomb substrate^1^,^2^,^3^,^4^,^5^. A cordierite (2MgO·2Al_2_O_3_·5SiO_2_) ceramic honeycomb is the most widely used substrate owing to its excellent thermal, chemical and mechanical properties^4^,^6^,^7^. Nevertheless, technological advances have led to increasing attention being paid to metallic substrates comprising thin Fe–Cr–Al alloy foils that are several tens of micrometres thick^4^,^8^,^9^. Their thinner walls offer the potential for higher cell densities with larger open frontal areas; consequently, lower pressure drops and the higher thermal conductivity of the abovementioned metallic substrates enables faster heating, which is especially important for the catalytic conversion of cold-start emissions. Conventional catalyst preparation uses similar wet impregnation and/or slurry coating processes regardless of the substrate material in order to deposit a porous washcoat layer on the substrate^4^. However, because the adhesion between a metal foil and the washcoat layer is less durable and the thermal expansion difference between them is large, frequent thermal and mechanical shocks can cause the washcoat layer to delaminate thus, serious catalyst deactivation may occur during long-term use^10^. Although this adhesion may be improved by surface pretreatment^11^,^12^,^13^,^14^, issues with coatability still limit the use of metallic honeycombs in automotive applications. Thus, the development of a more robust structure for metallic honeycomb catalysts remains a challenge^15^.

The aim of this study was to prepare novel metal honeycomb catalysts by applying the pulsed cathodic arc-plasma (AP) technique^16^,^17^,^18^,^19^,^20^. This simple but innovative approach enables the one-step deposition of catalytically active nanoparticles or nanofilms onto the surface of metal foils, as illustrated in Fig. 1a. By igniting pulsed discharges between a cathode and an anode, the metal on the cathode surface is vaporised and ionised and the ejected plasma collides with the substrate and deposits the metal nanoparticles. One of the key benefits of this technique is the uniform size and high catalytic activity of the as-prepared nanoparticles. This allows it to be employed for one-step, dry catalyst preparation, which is much more convenient than conventional wet processes that comprise a number of complex steps. Although several recent catalyst preparation studies have employed the AP technique to fabricate powder systems^16^,^17^,^18^,^19^,^20^, it is more suitable for two-dimensional metal substrates, such as Fe–Cr–Al foils, because of the very strong adhesion between the active metal and the metal substrate. For practical production, the continuous deposition of the catalyst is possible by the use of a roll-to-roll mechanism.

Among the various metal catalyst candidates assayed in our preliminary investigation, AP-deposited Rh was found to be the most active in simulated TWC reactions. Rh is widely used in TWC for NO_*x*_ purification because of its high efficiency towards NO dissociation^21^,^22^,^23^,^24^,^25^,^26^,^27^,^28^,^29^,^30^, but is the most scarce PGM. Increasing attention has therefore shifted towards the novel catalyst design that enables less amounts of Rh^31^,^32^,^33^,^34^,^35^,^36^. The present Rh overlayer catalyst achieved a higher specific activity using a lower Rh loading, compared with conventional supported Rh catalyst powders. Consequently, the structure of the Rh overlayer was thoroughly characterised to elucidate the reason for its high catalytic activity. Finally, the as-prepared metal foil catalysts were used to fabricate a monolithic honeycomb structure in order to evaluate their TWC performance under realistic gas composition and space velocity conditions.

## Results

### Structure of the Rh/stainless steel (SUS) foil catalyst prepared by AP

**Figure 1:**
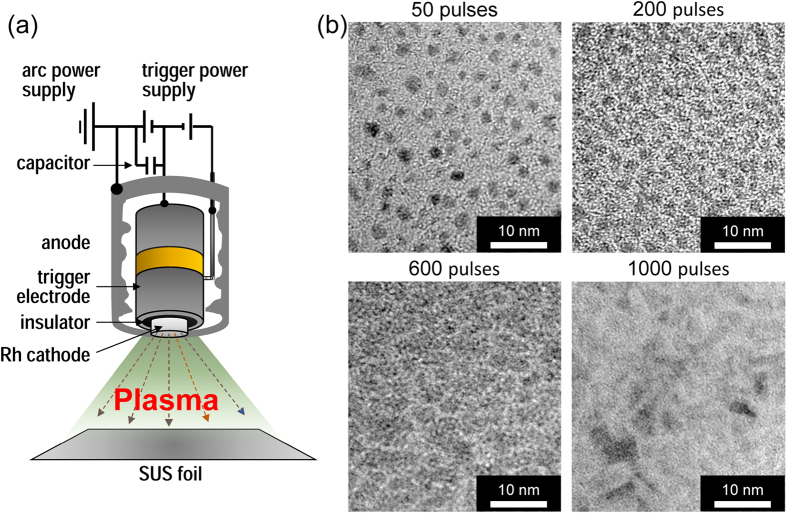
(**a**) Schematic of the pulsed AP deposition of Rh onto an SUS foil. (**b**) TEM images of Rh deposited with different numbers of AP pulses.

**Figure 2:**
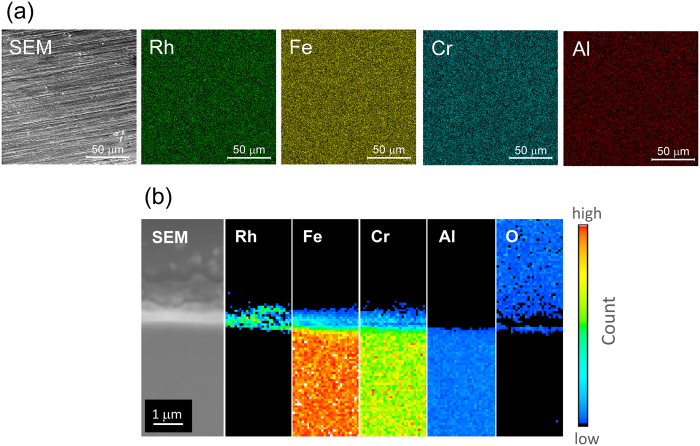
SEM and element mapping images of Rh/SUS. (**a**) Surface and (**b**) fracture surface views.

The microstructures of the Rh deposits prepared by the AP method were characterised by transmission electron microscopy (TEM). Figure 1b shows the TEM images of Rh as-deposited on a TEM grid covered with microgrid carbon films after different numbers of AP pulses. When 50 AP pulses are employed, highly dispersed Rh nanoparticles with a very narrow size distribution ranging from 1 to 3 nm (average size: 2.4 ± 1.1 nm) are observed. With an increasing number of pulses (200 and 600), the density of the nanoparticles increases, leading to coalescence and grain growth. After 1,000 AP pulses, nanoparticles are no longer observed and instead, a two-dimensional Rh overlayer is presented. Similar deposition behaviour is observed for the surface of an SUS foil, as can be seen by a scanning electron microscope (SEM) and X-ray mapping images of the top surface and the cross-sectional surface, as shown in Fig. 2. Rh is distributed uniformly on the top surface and does not penetrate into the SUS foil. The as-deposited Rh overlayer exhibits intimate contact with the surface of the SUS foil and is thus strongly adhered. A local structure analysis of Rh by Rh K-edge extended X-ray absorption fine structure (EXAFS) indicates that the Rh exists in its metallic form and that no alloys or compounds are formed on the surface of the SUS foil (see Supplementary Information, Figure S1 and Table S1).

**Figure 3:**
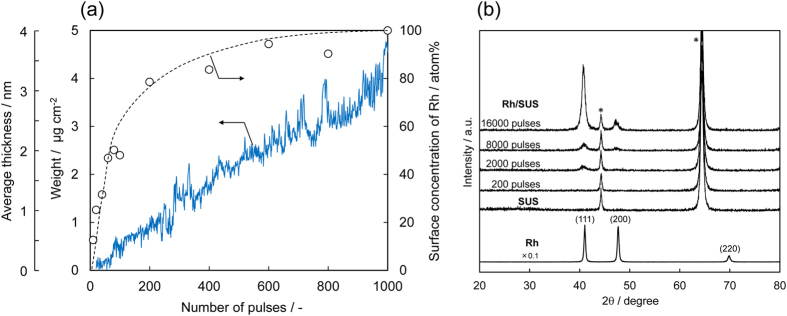
(**a**) (blue line) Weight, average thickness and (white circles) surface concentration of Rh as a function of number of AP pulses. (**b**) XRD patterns of the as-prepared Rh/SUS with different numbers of AP pulses. Peaks with an asterisk are from the SUS foil.

Figure 3a shows a plot of the weight of the Rh deposit per unit area of the SUS foil, the average thickness of the Rh overlayer and the surface concentration of Rh as a function of the number of AP pulses. The weight of the Rh deposit, as monitored by a quartz crystal microbalance, increases almost linearly with increasing pulse number. The Rh metal loading can therefore be controlled by varying the number of AP pulses. Conversely, the surface concentration of Rh, as determined by X-ray photoelectron spectroscopy (XPS), increases rapidly during the initial 200 AP pulses and reaches almost 100% after 1,000 pulses, where the deposit density reaches ca. 4 μg Rh cm^−2^. The XPS Rh3d binding energies (Rh3d_5/2_: 307.4 eV, Rh3d_3/2_: 312.2 eV) indicate that the surface Rh is metallic. Assuming a uniform thickness for the metallic Rh surface overlayer, its thickness was calculated to be 3.2 nm (Fig. 3a). Thus, the surface of the SUS foil is fully covered by a nanometric overlayer of Rh after 1,000 AP pulses. The full coverage of the SUS foil is unchanged by further AP pulsing, which simply causes an increase in the thickness of the Rh overlayer. Furthermore, the overlayer structure and its metallic state are stable during the catalytic reactions described below. Figure 3b shows the X-ray diffraction (XRD) patterns of the as-prepared Rh/SUS with different numbers of AP pulses. With an increased number of pulses, three peaks, which are ascribed to the (111), (200) and (220) reflections of metallic Rh, intensify. However, the (200) and (220) peaks are much less intense than the (111) peak compared with the relative peak intensities of a pure Rh metal foil reference. This indicates the (111) orientation of the as-deposited Rh overlayer along the SUS foil surface. A plausible reason for the Rh overlayer exposing this plane is its low surface energy and it is not considered to be the result of specific interactions with the SUS foil surface.

### Catalytic performance of Rh/SUS foil catalysts prepared by AP

**Figure 4:**
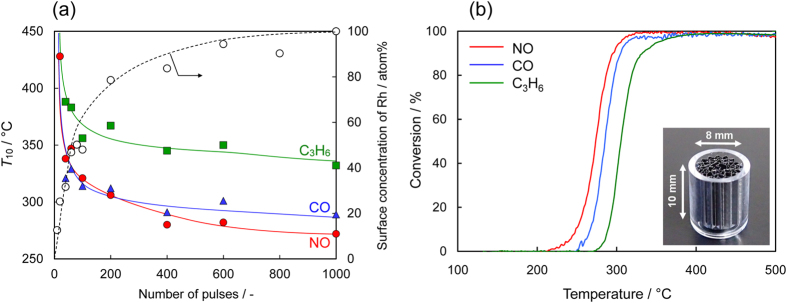
(**a**) (White circles) Rh surface concentration and light-off temperatures (*T*_10_) of NO, CO and C_3_H_6_ for the NO–CO–C_3_H_6_–O_2_ reaction over Rh/SUS foil catalysts prepared with different numbers of AP pulses. (**b**) Light-off curves of NO, CO and C_3_H_6_ in a simulated TWC reaction over a miniature honeycomb catalyst consisting of 1,000-pulse Rh/SUS foils. GHSV = 1.2 × 10^5^ h^−1^.

The catalytic activities of 2 mm × 30 mm Rh/SUS foil samples were obtained for the stoichiometric NO–CO–C_3_H_6_–O_2_ reaction in a flow reactor. Here, Rh was deposited on only one side of the SUS foils. The catalytic conversion efficiencies for NO, CO and C_3_H_6_ are strongly dependent on the number of pulses, i.e. the Rh loading (see Supplementary Information, Figure S2). Although the catalytic reaction initiates over the material prepared with just 40 AP pulses, the conversions of NO, CO and C_3_H_6_ do not reach completion, even at a reaction temperature of 500 °C. The light-off (i.e. a steep rise in conversion to ca. 100%) occurs over catalysts prepared with 100 or more AP pulses. Figure 4a plots the light-off temperature (*T*_10_; the reaction temperature at which the conversion reaches 10%) versus the number of AP pulses. The bare SUS foil shows negligible catalytic activity up to 500 °C; therefore *T*_10_ is significantly reduced by Rh deposition. The *T*_10_ value is lowered monotonically with an increase in AP pulse number in accordance with the increase in surface coverage by Rh. This indicates that higher surface coverage by Rh is beneficial for the light-off of TWC reactions. Notably, when the SUS foil is fully covered by other precious metals, such as Pt and Pd, the light-off cannot be observed and the NO conversion is less than 60%, even at 500 °C. The almost simultaneous light-off for NO, CO and C_3_H_6_ conversion is therefore attained over Rh/SUS foils only.

Finally, a miniature metal honeycomb was fabricated using flat and corrugated Rh/SUS foils (1,000 pulses of Rh deposition onto both sides), as shown in the inset of Fig. 4b, to evaluate their catalytic performance. A stoichiometric simulated exhaust gas was supplied at a gaseous hourly space velocity (GHSV) of 1.2 × 10^5^ h^−1^, which is close to that of practical TWC conditions. The honeycomb catalyst demonstrates steep light-off above 230 °C and almost complete conversion thereafter. This simulated TWC reaction involves a number of parallel elementary reactions including CO–O_2_, NO–CO, C_3_H_6_–O_2_, C_3_H_6_–NO, CO–H_2_O and C_3_H_6_–H_2_O. Judging from Fig. 4b, the light-off for NO occurs at the lowest temperature, followed by CO and subsequently C_3_H_6_. This sequence is in accordance with the catalytic behaviour for each elementary reaction; the catalyst is most active for NO–CO reactions. Although the activity is also high for the CO–O_2_ reaction, this reaction seems to be suppressed in the presence of NO. In contrast, the catalyst is slightly less active towards the reactions involving C_3_H_6_.

## Discussion

The present results demonstrate that Rh overlayers formed on the surface of SUS foils have the potential for TWC performance under practical space velocity conditions despite their nonporosity and small surface area, which is in complete contrast to conventional porous powdered catalysts. Note that other PGMs, such as Pt and Pd, are much less active than Rh when they are coated onto SUS foils by the AP deposition technique. This seems to be inconsistent with the fact that PGM nanoparticle catalysts, especially Pd, supported on high-surface area porous support materials, such as Al_2_O_3_, ZrO_2_ and CeO_2_, possess TWC activities comparable with those of supported Rh catalysts^37^,^38^,^39^. These results suggest that size and/or geometric effects are important factors depending on the metal elements. To further explore this idea, it is necessary to compare the present Rh overlayer catalyst with reference catalysts, i.e. a pure Rh metal foil and a conventional supported Rh nanoparticle catalyst.

Table 1 shows the turnover frequency (TOF) for NO conversion in simulated TWC reactions over a 1,000-pulse Rh/SUS foil (2 mm × 30 mm, full coverage on one side only) and a pure Rh metal foil with similar dimensions (2 mm × 30 mm × 0.2 mm). According to surface roughness analysis using a confocal laser scanning microscope, the true surface areas of these samples are nearly equal to (i.e. 1.013-fold higher than) the geometric surface areas. As indicated by XRD measurement (Fig. 3b), the present Rh overlayer shows an approximately (111) orientation. It is not thought that the overlayer is exactly modelled by the (111) surface; the surface structure is undoubtedly more complex, indicated by its root mean square surface roughness of ca. 50 nm. However, the number of surface Rh sites was estimated from the geometric area of the foils assuming exposure of the (111) surface, as shown in Table 1. Because both samples exhibit similar light-off curves (see Supplementary Information, Figure S3), the TOF was calculated from the NO conversion in the NO–CO–C_3_H_6_–O_2_ reaction below 20% (at 260 °C), which allows a rough approximation for a differential reactor. The resulting TOF over Rh/SUS (74.1 min^−1^) is roughly 5-fold greater than that over the Rh metal foil (15.8 min^−1^). More interestingly, the TOF over Rh/SUS is more than 40-fold greater than that over the conventional supported nanoparticle catalyst (50 mg of 0.4 mass% Rh/ZrO_2_). Considering the Rh content (200 μg), the Rh metal dispersion measured by CO chemisorption (17.5%) and the NO conversion at 260 °C (24%), the TOF for Rh/ZrO_2_ is calculated to be ca. 1.6 min^−1^. This is close to the value previously reported for 0.5 mass% Rh/Al_2_O_3_ (3.1 min^−1^)^40^, although the reaction temperature (230 °C) and gas composition (1% CO, 0.5% NO) were different from those of the present study.

A similar comparison was made between honeycomb catalysts consisting of Rh/SUS and a reference Rh/ZrO_2_/cordierite. As indicated in Fig. 4b, the present Rh/SUS is most efficient for the NO–CO reaction. Therefore, to simplify the discussion, we focused on this reaction to elucidate how the Rh/SUS foil catalyst is different from the conventional powdered catalysts. The bottom part of Table 1 shows the TOFs for the stoichiometric NO–CO reaction over the two honeycomb catalysts (see Figure S4, Supplementary Information, for the light-off data). The Rh/ZrO_2_/cordierite honeycomb contained 50 mg of the catalyst powder (0.4 mass% Rh/ZrO_2_) and thus 200 μg of Rh. The TOF for NO conversion was thus calculated to be ca. 1.05 min^−1^ at 295 °C. Conversely, the Rh/SUS honeycomb, which was prepared from double-sided 400-pulse foils, contained 34.2 μg of Rh (ca. 1/6 that of the reference catalyst) with 90% coverage of the total area of the foils (10 mm × 190 mm). Again, assuming that Rh(111) is exposed on the SUS surface, the number of surface Rh sites is estimated to be 45.5 nmol. Using this value and a NO conversion at 295 °C of 17.5%, the TOF is calculated to be 86.0 min^−1^, which is more than 80-fold greater than that of the Rh/ZrO_2_/cordierite honeycomb catalyst (1.05 min^−1^). Consequently, the high TOF for the NO–CO reaction over the Rh overlayer plays a key role in effecting adequate TWC performance under realistic reactant composition and space velocity conditions (Fig. 4b).

It is well-known that NO dissociation into atomic N and O is a crucial step in the NO–CO reaction^41^,^42^,^43^. To further discuss the contribution of the Rh overlayer to this reaction, the surface structure sensitivity of NO dissociation over Rh, which has been described in the literature by several researchers, should be summarised. Many surface science studies employing well-defined Rh surfaces have confirmed that Rh crystal faces with lower coordination numbers are more active for NO dissociation^21^,^22^,^23^,^24^,^26^,^44^,^45^,^46^. This means that surfaces with more open faces, like Rh(110), tend to be more active than Rh(111)^22^,^24^,^26^,^46^. More interestingly, it has been confirmed that NO dissociation, and thus the NO–CO reaction, on single-crystal Rh proceeds much faster than on supported Rh nanoparticles^3^,^40^. More direct evidence of this phenomenon was provided by Oh and Eickel^40^, who reported that the TOF for the NO–CO reaction increased with an increase of the Rh particle size, with a 45-fold higher TOF being obtained as the Rh particle size increased from 1.0 to 67.6 nm. Furthermore, the NO–CO reaction has been reported to proceed nearly 40 times faster over single-crystal Rh(111) than over Rh/Al_2_O_3_ powder catalysts at 350 °C^47^. Such a size dependence is explained by the slower dissociation of adsorbed NO, or by the suppression of N_2_ desorption via NO_*s*_ + N_*s*_ → N_2_ + O_*s*_ (“_*s*_” means surface species) with smaller Rh particles^40^,^47^. Peden *et al*. reported that large Rh particles (more than 28 nm in size) appeared to have Rh(110)-like higher TOFs for the NO–CO reaction, while small Rh particles tended to show lower TOFs similar to those for Rh(111)^28^. The difference between Rh(110) and (111) is associated with steric crowding of the adsorbed NO, which inhibits NO dissociation and leads to lower N atom coverage and thus lower N_2_ formation.

These previous studies suggest that the loss of Rh metal surface area resulting from the particle size may be largely offset by the attendant increase in TOF. Unfortunately, catalyst design based on this concept has not been realistic for three-dimensional structures due to the scarcity of Rh and thus its high cost. In contrast, an SUS foil fully covered by a two-dimensional Rh overlayer with a nanometric thickness is a promising alternative structure because of its high catalytic performance at lower Rh loading levels. The present Rh/SUS foil shows a nearly 80-fold higher TOF than the Rh/ZrO_2_ powder (Table 1), with the difference being comparable with those observed in the previous studies described above. High overall rates for the NO–CO reaction are superior to those of the reference powder catalyst (Rh/ZrO_2_), which contains more than six times the amount of Rh. It is also noteworthy that the TOF for the NO–CO–C_3_H_6_–O_2_ reaction is still five times higher over the Rh/SUS foil than over pure Rh metal foil. While the present study has not elucidated a detailed mechanism for the reaction over the SUS foil-supported Rh overlayer, it has provided important new information on its extremely high TOF in the NO–CO reaction and in simulated TWC reactions. The concept of overlayer catalysts can be widely extended to the design and production of new metal honeycomb catalysts without the need for conventional wet impregnation and slurry coating. In this regard, the AP catalyst preparation technique is beneficial for the production of thin overlayer catalysts on metallic foil substrates, which affords novel and interesting catalysts capable of practically meaningful performance.

## Conclusions

The AP deposition of metallic Rh onto an SUS foil was found to be a novel and efficient preparation process for a catalytic TWC converter material. The (111)-oriented and ca. 3 nm-thick Rh overlayer, which fully covered the surface of the SUS foil, was obtained after 1,000 AP pulses. Honeycomb catalysts fabricated from flat and corrugated Rh/SUS foils successfully demonstrated light-off in simulated TWC reactions under practical space velocity conditions (GHSV = 1.2 × 10^5^ h^−1^). Compared with a conventional Rh/ZrO_2_/cordierite honeycomb, which was prepared by wet impregnation and slurry coating, the Rh/SUS honeycomb achieved faster light-off of the vital NO–CO reaction and consequently achieved a ca. 80-fold higher TOF. Despite its small number of active sites in comparison with the conventional supported nanoparticle powder catalysts, the very high TOF of the Rh overlayer enabled high overall reaction rates using a lower Rh loading.

## Methods

### Catalyst preparation and characterisation

As shown in Fig. 1a, the AP deposition apparatus consisted of a vacuum chamber with a turbo-molecular pumping system, an arc discharge source (Ulvac Inc., ARL-300) with a Rh metal cathode (ϕ 10 mm, 99.9%, Furuya Metals, Co. Ltd.) and a rotating container for substrates. A commercially available Fe–Cr–Al SUS foil (75 atom% Fe, 20 atom% Cr, 5 atom% Al, Nippon Steel & Sumikin Materials) with a thickness of 51 μm was placed below the AP source in the vacuum chamber. A pulsed arc-plasma with a period of 0.2 ms and a current amplitude of 2 kA was generated from a Rh metal target with a frequency of 1.0 Hz and was irradiated onto an SUS foil to deposit Rh at ambient temperature. The Rh loading on the SUS foil was determined using a quartz crystal microbalance (STM-2, Inficon). As-prepared Rh-coated foils are expressed in terms of Rh/SUS. Flat and corrugated foils (10 mm × 40 mm and 10 mm × 55 mm, respectively) after 400 AP pulses on each side were wrapped together into a monolithic honeycomb shape and fixed in a quartz tube (ϕ 10 mm × 10 mm, 600–800 cells in^−2^, see the inset of Fig. 4b). A reference honeycomb catalyst with a similar geometry was prepared by a conventional wet process. A powdered catalyst, 0.4 mass% Rh/ZrO_2_, was prepared by the impregnation of ZrO_2_ powders (JRC-ZRO-3, the Catalysis Society of Japan) with an aqueous solution of Rh(NO_3_)_3_ (Tanaka Kikinzoku), followed by drying and calcining at 600 °C for 3 h in air. The reference honeycomb catalyst was prepared by dipping a cordierite honeycomb (ϕ 10 mm × 10 mm, 600 cells in^−2^, NGK Insulators, Ltd.) into a slurry, which was prepared by ball-milling of the catalyst powder (Rh/ZrO_2_), an inorganic binder and water, followed by drying at 90 °C and calcination at 600 °C for 3 h.

The characterisation of Rh/SUS foil samples was conducted using X-ray diffraction XRD, XPS, EXAFS, TEM, SEM and laser microscopy. The crystal structure of the sample was determined using XRD employing monochromatic CuKα radiation (40 kV, 200 mA, RINT2000, Rigaku). The surface analysis of the sample was conducted with XPS using monochromated AlKα radiation (12 keV, K-Alpha, Thermo Fisher Scientific). The binding energy was charge-referenced to C 1s at 285 eV. The local coordination around the Rh atoms was investigated by Rh K-edge EXAFS, which was recorded on an NW10A station at the Photon Factory Advanced Ring, High Energy Accelerator Research Organization (KEK) (Proposal No. 2014G567). The spectral data were recorded at room temperature in transmission mode using a Si(311) double-crystal monochromator, or in fluorescence mode using a solid-state detector. TEM and SEM micrographs were acquired using an FEI TECNAI F20 operating at 200 kV or an FEI QUANTA FEG 250 operating at 20 keV, respectively. For elemental mapping analysis, energy-dispersive X-ray analysis (TEAM, EDAX) was used. The surface topographic analysis was performed by a confocal laser scanning microscope (VK-X250, Keyence).

### Catalytic reactions

The catalytic light-off performance was evaluated in a flow reactor. For this purpose, SUS foils after different numbers of AP pulses were cut into rectangular strips with dimensions of 2 mm × 30 mm, and fixed in a quartz tube with an inner diameter of 4 mm. A high-purity Rh metal foil (99.9%, Furuya Metals, Co. Ltd.) with similar dimensions (2 mm × 30 mm × 0.2 mm) and a powder catalyst (0.4 mass% Rh/ZrO_2_, 50 mg) were used as reference catalysts. The number of surface Rh sites for the foil samples was calculated using geometric areas. For the pure Rh metal foil, the edge areas accounted for 9.6% of the total area. The number of surface Rh sites for the powder catalyst was determined by CO chemisorption measurement at 50 °C based on 2:1 stoichiometry between chemisorbed CO and surface Rh. A stoichiometric gas mixture containing 0.05% NO, 0.51% CO, 0.04% C_3_H_6_ and 0.4% O_2_ and a He balance was supplied at 100 mL min^−1^ during heating. The gas concentration was analysed using a mass spectrometer (GSD30301, Pfeiffer) and nondispersive infrared detectors (VA3000, Horiba). For the honeycomb-shaped catalysts, a simulated TWC reaction was performed using a Horiba SIGU/MEXA reaction analysis system, which consisted of a gas supply unit, an infrared image furnace with four ellipsoidal reflectors and a gas analysis unit. The catalytic activity was evaluated by heating the catalyst bed from 100 to 500 °C at a rate of 10 °C min^−1^ while supplying the simulated stoichiometric gas mixture (0.05% NO, 0.50% CO, 0.05% C_3_H_6_, 0.53% O_2_, 0.17% H_2_, 10% CO_2_, 10% H_2_O, and N_2_ balance, 1.0 L min^−1^, GHSV = 1.2 × 10^5^ h^−1^) at atmospheric pressure. The NO–CO reaction was also performed in a similar way (0.05% NO, 0.05% CO, N_2_ balance, 1.0 L min^−1^, GHSV = 1.2 × 10^5^ h^−1^). The gas analysis was performed by nondispersive infrared CO detectors, a flame ionisation hydrocarbon detector, a chemiluminescence NO detector and a magnetopneumatic O_2_ detector.

## Additional Information

**How to cite this article**: Misumi, S. *et al*. A nanometric Rh overlayer on a metal foil surface as a highly efficient three-way catalyst. *Sci. Rep.* **6**, 29737; doi: 10.1038/srep29737 (2016).

## Supplementary Material

Supplementary Information

## Figures and Tables

**Table 1:** Activity comparison between Rh/SUS and Rh/ZrO_2_/cordierite honeycombs.

catalyst	substrate	Rh loading/μg	surface Rh/μmol	NO conv.^a^/%	TOF/min^−1^
Rh overlayer (1,000 pulses, 2 mm × 30 mm)	SUS foil strip	2.7	0.0016^a^	5.3^c^	74.1
Rh foil (2 mm × 30 mm)	–	73.1 × 10^3^	0.0035^a^	2.5^c^	15.8
Rh/ZrO_2_ powder (0.4 mass%, 50 mg)	–	200	0.340^b^	24.0^c^	1.6
Rh overlayer (400 pulses, 10 mm × 190 mm)	SUS foil honeycomb	34.2	0.0455^a^	17.5^d^	86.0
Rh/ZrO_2_ powder (0.4 mass%, 50 mg)	cordierite honeycomb	200	0.340^b^	1.6^d^	1.05

^a^Determined by the geometric area of a foil surface, surface coverage and atomic density of the Rh(111) (1.60 × 10^19^ atom m^−2^).

^b^Determined by the Rh loading (0.20 g) and Rh metal dispersion (17.5%).

^c^NO conversion for the NO–CO–C_3_H_6_–O_2_ reaction (0.05% NO, 0.5% CO, 0.04% C_3_H_6_, 0.4% O_2_, He balance, 0.1 L min^−1^, 260 °C).

^d^NO conversion for the NO–CO reaction (0.05% NO, 0.05% CO, N_2_ balance, 1.0 L min^−1^, GHSV = 1.2 × 10^5^ h^−1^ (Rh/SUS) or 0.76 × 10^5^ h^−1^ (Rh/ZrO_2_), 295 °C).
