# Supplementary material for: A nanometric Rh overlayer on a metal foil surface as a highly efficient three-way catalyst
Source: Sci Rep. 2016 Jul 8;6:29737. doi: 10.1038/srep29737 (PMC4937386; doi:10.1038/srep29737)
Supplement: Supplementary Information [file srep29737-s1.pdf]

Supplementary Information

A nanometric Rh overlayer on a metal foil surface as a highly efficient three-way catalyst

Satoshi Misumi,[1] Hiroshi Yoshida,[1,2] Satoshi Hinokuma,[1,2,3] Tetsuya Sato,[4] & Masato Machida[1,2,†]

[1] Department of Applied Chemistry and Biochemistry, Graduate School of Science and Technology, Kumamoto University, Kumamoto, 860-8555, Japan

[2] Unit of Elements Strategy Initiative for Catalysts & Batteries, Kyoto University, Kyoto, 615-8245, Japan

[3] Precursory Research for Embryonic Science and Technology, Japan Science and Technology Agency, Saitama, 332-0012, Japan

[4] Technical Division, Faculty of Engineering, Kumamoto University, Kumamoto, 860-8555, Japan

Figure S1 Fourier-transformed Rh K-edge EXAFS for an as-prepared Rh/SUS foil and Rh metal as a reference.

Figure S2 Light-off curves for the stoichiometric NO–CO–C_3H_6–O_2 reaction for 2 mm × 30 mm Rh/SUS foil catalysts prepared with different numbers of AP pulses.

Figure S3 Light-off curves for stoichiometric NO–CO–C_3H_6–O_2 reaction for (solid lines) a 1,000-pulse Rh/SUS foil and (dotted lines) a pure Rh metal foil, both with a size of 2 mm × 30 mm.

Figure S4 Light-off curves for the stoichiometric NO–CO reaction over honeycomb catalysts. Solid lines: 400-pulse Rh/SUS foil. Dotted lines: 0.4 mass% Rh/ZrO_2/cordierite.

Table S1 The fitting parameters obtained from Rh K-edge EXAFS analysis.

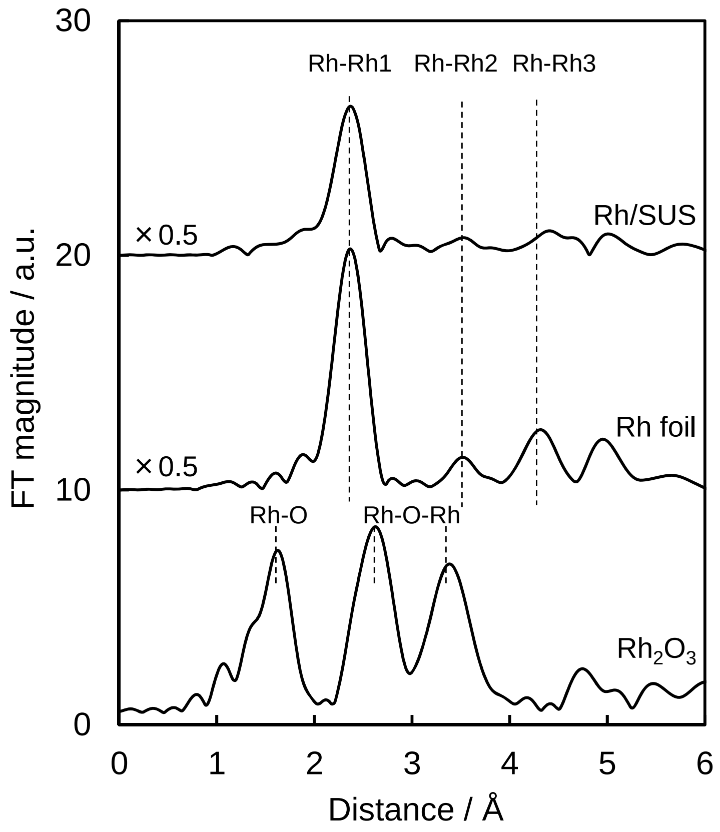

Figure S1 Fourier transformed Rh K-edge EXAFS for as-prepared Rh/SUS foil, Rh metal foil and Rh_2O_3 as references. See Table S1 for curve fitting analysis results.

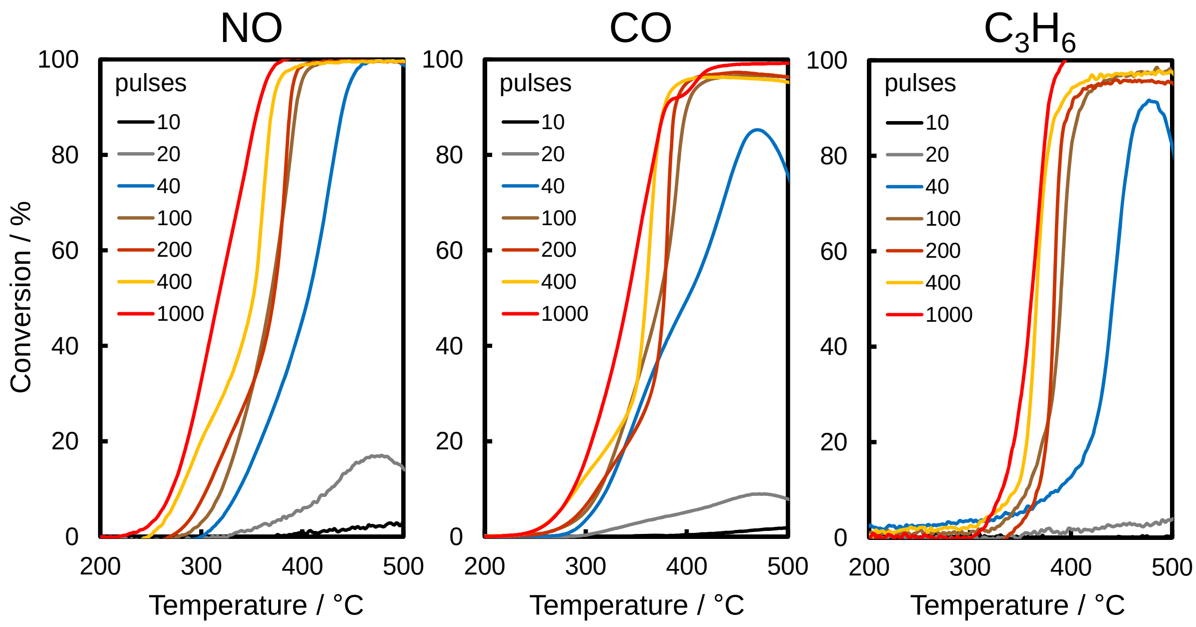

Figure S2 Light-off curves for stoichiometric NO−CO−C_3H_6−O_2 reaction for Rh/SUS foil catalysts with a size of 2 mm×30 mm prepared with different numbers of AP pulsing.

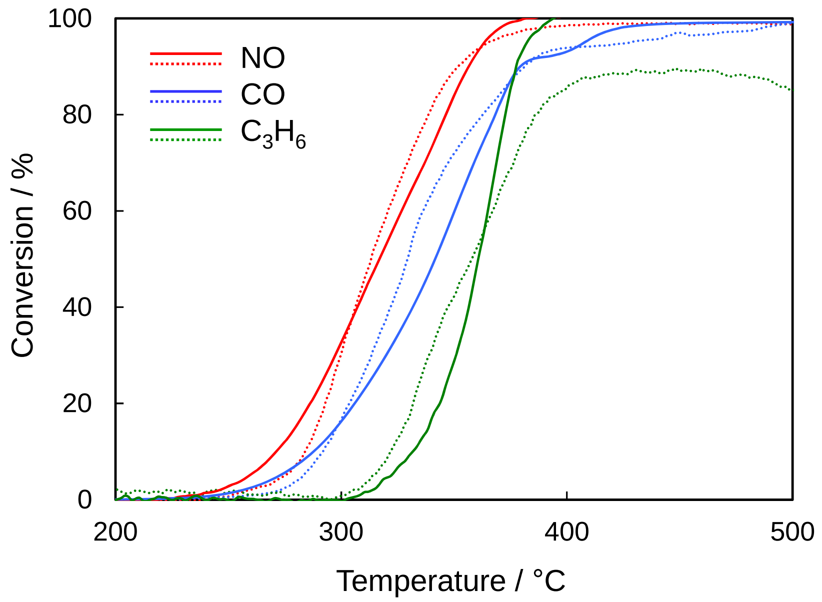

Figure S3 Light-off curves for stoichiometric NO−CO−C_3H_6−O_2 reaction for (solid lines) 1000 pulses Rh/SUS foil and (dotted lines) pure Rh metal foil with a size of 2 mm×30 mm. 0.05% NO, 0.5% CO, 0.04% C_3H_6, 0.4% O_2, He balance.

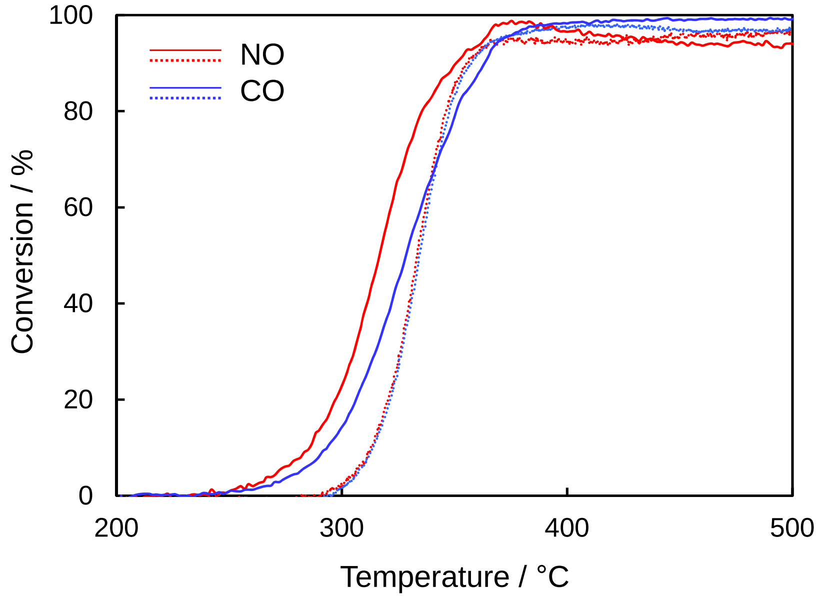

Figure S4 Light-off curves for stoichiometric NO−CO reaction over honeycomb catalysts. Solid lines: 400 pulses Rh/SUS foil. Dotted lines: 0.4 mass% Rh/ZrO_2/cordierite. 0.05% NO, 0.05% CO, N_2 balance. balance, 1 L min^{-1}, GHSV=1.2×10^5 h^{-1} (Rh/SUS) or 0.76×10^5 h^{-1} (Rh/ZrO_2).

Table S1 The fitting parameters obtained from Rh K-edge EXAFS analysis (Figure S1)

Samples	Shell	r/Å [a]	±	CN [b]	±	$\sigma^2/10^{-2}$ Å^2 [c]	±	R/%
Rh/SUS [d]	Rh–Rh1	2.69	0.01	8.0	1.0	0.52	0.1	1.2
	Rh–Rh2	3.81	0.03	3.8	2.5	0.74	0.3	
	Rh–Rh3	4.72	0.01	4.2	2.4	0.38	0.2	
Rh foil [e]	Rh–Rh1	2.69	0.01	12.0	-	0.44	0.1	3.6
	Rh–Rh2	3.83	0.03	6.0	-	0.60	0.1	
	Rh–Rh3	4.67	0.01	24.0	-	0.63	0.1	
Rh_2O_3 [e]	Rh–O	2.04	0.01	6.0	-	0.40	0.1	4.5
	Rh–O–Rh	2.99	0.02	3.0	-	0.55	0.1	
	Rh–O–Rh	3.52	0.01	3.0	-	0.43	0.1	

Interval of k-space to r-space of FT is 2.0–17.0 Å^{-1}.

[a] Atomic distance.

[b] Coordination number.

[c] Debye–Waller factor.

[d] Measured in transmission mode.

[e] Measured in fluorescence mode.
